# Bioinformatics and system biology analysis revealed the crosstalk between COVID‐19 and osteoarthritis

**DOI:** 10.1002/iid3.1123

**Published:** 2023-12-22

**Authors:** Bowen Lai, Heng Jiang, Taotao Liao, Yuan Gao, Xuhui Zhou

**Affiliations:** ^1^ Department of Orthopedics Changzheng Hospital, Second Military Medical University Shanghai China

**Keywords:** bioinformatics, coronavirus disease 2019, drug therapy, hub genes, immune infiltration, osteoarthritis

## Abstract

**Background:**

The global coronavirus disease 2019 (COVID‐19) outbreak has significantly impacted public health. Moreover, there has been an association between the incidence and severity of osteoarthritis (OA) and the onset of COVID‐19. However, the optimal diagnosis and treatment strategies for patients with both diseases remain uncertain. Bioinformatics is a novel approach that may help find the common pathology between COVID‐19 and OA.

**Methods:**

Differentially expressed genes (DEGs) were screened by R package “limma.” Functional enrichment analyses were performed to find key biological functions. Protein–protein interaction (PPI) network was constructed by STRING database and then Cytoscape was used to select hub genes. External data sets and OA mouse model validated and identified the hub genes in both mRNA and protein levels. Related transcriptional factors (TF) and microRNAs (miRNAs) were predicted with miRTarBase and JASPR database. Candidate drugs were obtained from Drug Signatures database. The immune infiltration levels of COVID‐19 and OA were evaluated by CIBERSORT and scRNA‐seq.

**Results:**

A total of 74 common DEGs were identified between COVID‐19 and OA. Receiver operating characteristic curves validated the effective diagnostic values (area under curve > 0.7) of four hub genes (matrix metalloproteinases 9, ATF3, CCL4, and RELA) in both the training and validation data sets of COVID‐19 and OA. Quantitative polymerase chain reaction and Western Blot showed significantly higher hub gene expression in OA mice than in healthy controls. A total of 84 miRNAs and 28 TFs were identified to regulate the process of hub gene expression. The top 10 potential drugs were screened including “Simvastatin,” “Hydrocortisone,” and “Troglitazone” which have been proven by Food and Drug Administration. Correlated with hub gene expression, Macrophage M0 was highly expressed while Natural killer cells and Mast cells were low in both COVID‐19 and OA.

**Conclusion:**

Four hub genes, disease‐related miRNAs, TFs, drugs, and immune infiltration help to understand the pathogenesis and perform further studies, providing a potential therapy target for COVID‐19 and OA.

## Graphical abstract

There exists a genetic and biological association between coronavirus disease 2019 (COVID‐19) and osteoarthritis (OA) λ matrix metalloproteinases 9, ATF3, CCL4, RELA are higher expressed in both COVID‐19 and OA λ Bioinformatics analysis identified potential diagnostic methods and therapeutic strategies toward the co‐morbidity of COVID‐19 and OA.

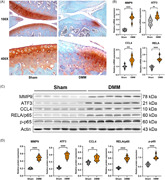

## 1 INTRODUCTION

Coronavirus disease 2019 (COVID‐19), caused by severe acute respiratory syndrome Coronavirus 2 (SARS‐CoV‐2), is a disease primarily associated with respiratory symptoms but can affect multiple organs and systems within the body.^1^, ^2^ With almost 1 billion people worldwide infected and the emergence of potential new mutant strains, the impact on society is significant, particularly on older individuals who are at higher risk of severe symptoms and mortality.^3^ Despite numerous clinical trials, effective drugs specifically for COVID‐19 are still lacking, making treatment difficult.

Osteoarthritis (OA) is a degenerative disease that affects large joints, causing joint pain and abnormal movement that can lead to disability in severe cases.^4^ With a prevalence of around 60% in hands, 33% in knees, and 5% in hips in individuals over 65 years of age,^5^ OA is one of the most common joint disorders. Studies have shed some light on the pathogenesis and biomarkers for the diagnosis and treatment of OA. However, there are still no specific diagnostic criteria or targeted drug therapy for OA‐related molecules. Given the inflammatory and immune disorders that both COVID‐19 and OA share, researchers have begun to explore the potential links between these two diseases. A cross‐sectional study among elders over 60 years old indicated a significant increase in the prevalence of OA during the COVID‐19 pandemic from 45.3% to 54.7%.^6^ Additionally, patients with OA show higher in‐hospital mortality than controls (odds ratio = 1.11).^7^ Researchers have speculated that angiotensin‐converting enzyme 2 and serine proteases may lead to cytokine storms and intense inflammation, increasing the risk of musculoskeletal diseases such as OA.^8^ However, no studies have yet explored the pathogenesis or treatment of comorbidity of COVID‐19 and OA.

**Figure 1:**
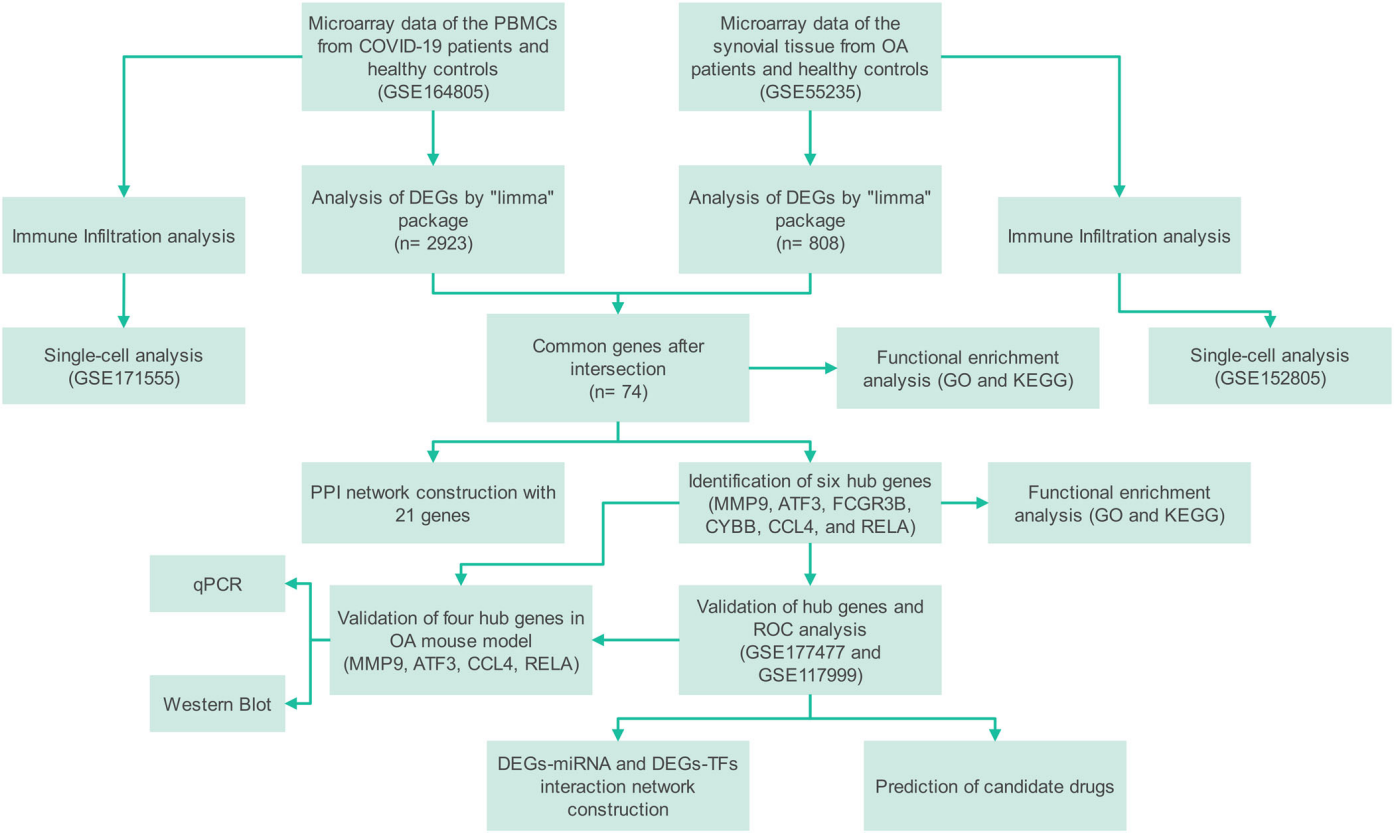
The flowchart of the study process.

Bioinformatics, using high‐throughput data, is a cross‐disciplinary tool that can aid in investigating disease pathophysiological mechanisms. Studies have found the potential genetic effects of COVID‐19 on idiopathic pulmonary fibrosis^9^ and pulmonary arterial hypertension^10^ patients by bioinformatics methods. In this study (Figure 1), we integrated sequencing data of COVID‐19 and OA from the Gene Expression Omnibus (GEO) database and identified common differentially expressed genes (DEGs) between the two diseases. Functional enrichment analysis was conducted to explore the biological processes involved in pathogenesis, followed by protein–protein interaction (PPI) network analysis to identify hub genes and their interaction with DEGs. We then validated the function of hub genes using receiver operating characteristic (ROC) curves, external data sets, and mouse models. Matrix metalloproteinases (MMP) 9, ATF3, CCL4, and RELA were identified as hub genes. The construction of the PPI network, gene–microRNA (miRNA) interaction, and gene–transcriptional factors (TFs) interaction can help the development of new therapeutic strategies in the levels of genes and proteins.^11^, ^12^, ^13^ Thus, we predicted links between TFs and miRNAs, identifying a total of 28 TFs and 82 miRNAs associated with the four hub genes that may influence the development of COVID‐19 and OA. Additionally, effective candidate drugs were screened that may be helpful for patients. Immune infiltration analysis identified the role of immune reactions in the diseases and the association between hub genes and immune cell expression levels. Our study offers a strong theoretical foundation for the future development of diagnostic methods and molecular therapies for the co‐occurrence of COVID‐19 and OA.

## 2 METHODS

### 2.1 Data source

The expression profiling by microarray in this study was obtained from the GEO database (https://www.ncbi.nlm.nih.gov/geo). GSE164805 contains the whole genome transcriptome to peripheral blood mononuclear cells (PBMCs) taken from COVID‐19 patients (*n* = 10) and healthy controls (*n* = 5). GSE55235 provides the microarray data from the synovial tissue of OA patients (*n* = 10) and healthy controls (*n* = 10). GSE177477 and GSE117999 were used as the external validation data sets for COVID‐19 and OA, respectively.

### 2.2 Identification of the overlapping DEGs between COVID‐19 and OA

We first use R packages “limma” and “affy”^14^ to normalize the expression matrix for the following analysis and evaluate the quality of data by R package “arrayQualityMetrics.”^15^ After normalization, We prefilter and remove the low count genes and use “limma” R package to identify the DEGs with the criteria of |log2 Fold change| > 1 and adjusted *p* value (Benjamini–Hochberg method) <.05. Finally, we draw the Venn diagrams to identify the common upregulated and downregulated genes by jvenn (http://jvenn.toulouse.inra.fr/app/index.html).^16^

### 2.3 Gene Ontology (GO) functions and Kyoto Encyclopedia of Genes and Genomes (KEGG) pathways enrichment analyses of DEGs

GO provides information on the functions of genes by classifying gene products into three separate categories: biological process, cellular component, and molecular function. KEGG is a database that can be used to deduce the high‐level utilities of a biological system from molecular‐level data, such as signaling pathways. Based on these two databases, functions and pathways enrichment analysis of DEGs and identified hub genes were identified and visualized by R package “clusterProfiler” with the criteria of *p* < .05.^17^

### 2.4 PPI network construction and identification of the hub genes

Interactions between proteins can often be inferred from genomic correlations between the genes that encode them. Thus, we established a PPI network with the identified DEGs using STRING (https://string-db.org). Proteins that combined scores >0.4 were involved in the PPI networks. Cytoscape (Version 3.9.1) was used to modify and beautify the network. Cytohubba, an app in Cytoscape, can predict and explore important nodes and subnetworks in PPI network by several topological algorithms.^18^ Maximal Clique Centrality (MCC) is an algorithm that can excavate the hub genes through network topology analysis. Genes with MCC scores >8 were identified as the hub genes and involved in the subsequent analysis.

### 2.5 ROC evaluation

The ROC curve can be used to determine the best diagnostic values of each gene. The true positive rate and sensitivity of the test increase with the proximity of the ROC curve to the upper left corner, but the false positive rate and misdiagnosis rates decrease, showing a stronger diagnostic value of the gene for the disease. R packages “pROC” was used to evaluate and draw the ROC curve. Area under curve (AUC) is the area enclosed by the ROC curve and the axis and the hub genes having an AUC of >0.7 were regarded as helpful in disease diagnosis.

### 2.6 Validation of hub genes in external data sets

The external data set of COVID‐19 (GSE177477) and OA (GSE117999) were used to validate the hub gene expression and diagnosis values. Blood mRNA transcriptomes from GSE177477 were determined in COVID‐19 cases (*n* = 29) and healthy controls (*n* = 18) while GSE117999 identified gene transcripts differentially expressed in cartilage tissues obtained from patients with OA (*n* = 12) and healthy controls (*n* = 12). Box plots were performed to visualize the different expressions of six hub genes in patients and the controls. The hub genes may be significant if the expression patterns were the same in both the external data set and the training data set. Moreover, we conduct the ROC evaluation and plot the curve to validate the diagnosis values of six hub genes in COVID‐19 and OA.

### 2.7 Animals and DMM‐induced OA mouse model

A total of 12 male 10‐week‐old C57BL/6 mice (weight 18–20 g) were purchased from specific pathogen‐fre (SPF) Biotechnology Co. Ltd. (Beijing, China) and kept in the Animal Experimental Center of Second Military Medical University under the SPF conditions. OA was induced by surgical destabilization of the medial meniscus (DMM) on the left knee joints of mice as previously described.^19^ Sham operation was performed by simple arthrotomy without the transaction of the medial meniscotibial ligament. After surgery, the mice were randomly divided into two groups (*n* = 6 in each), the Control group (sham‐operated) and the OA group (DMM surgery). Four weeks after the DMM surgery, the mice in each group were killed and the knee joints were collected for further analysis. All experiments were conducted with the guidelines of the Ethics Committee on Animal Experiments of the Second Military Medical University.

### 2.8 Histopathologic analysis

The mice were killed by an overdose of 1% pentobarbital sodium injection, and the knee joints were harvested 4 weeks after surgery. After being fixed in 4% paraformaldehyde for 24 h and decalcified in 10% ethylene diamine tetraacetic acid, the specimens were embedded in paraffin and 5‐μm sections. Slides of each joint sample were stained with safranin O‐fast green.

### 2.9 Quantitative polymerase chain reaction (qPCR)

The total RNA was extracted from mice articular cartilage of the Control group and the OA group (each with six biological replicants) using TRIZOL reagent (Invitrogen). The SuperScript III First‐Strand Synthesis SuperMix (Invitrogen) was used to convert mRNA to cDNA. All reactions were run on a StepOne Real‐time PCR System (Applied Biosystems). ΔCt was calculated by deducting the target genes' Cycle threshold (*C*_t_) values from the GAPDH's. The results were evaluated using the $2^{‐∆∆C_{t}}$ method. The primer sequences used were as follows: GAPDH (F: 5′‐CGGCAAATTCAACGGCACAG‐3′, R: 5′‐GACATACTCAGCACCGGCCTCA‐3′); MMP9 (F: 5′‐TCTACACGGAGCACGGCAAC‐3′, R: 5′‐ACCAGCGGTAACCATCCGA‐3′); ATF3 (F: 5′‐AGCTGAGATTCGCCATCCA‐3′, R: 5′‐GCCTCAGACTTGGTGACTGAC‐3′); CCL4 (F: 5′‐CCAATGGGCTCTGACCCTC‐3′; R: 5′‐CTGGCTTGGAGCAAAGACTG‐3′); RELA (F: 5′‐CGAGCTCAAGATCTGCCGAGT‐3′; R: 5′‐CACAGCAAGAAGATCTCATCCCC‐3′).

### 2.10 Western blot

The articular cartilage of mice was washed in ice‐cold PBS and incubated in radioimmunoprecipitation assay. Bicinchoninic acid protein assay kit (MDL) was used to measure the protein concentrations. Then, 20 ug protein of each sample was loaded in sodium dodecyl sulfate polyacrylamide gel electrophoresis precast gel (MDL) and transferred to a polyvinylidene fluoride membrane. After blocked, the membranes were incubated overnight with anti‐β‐actin (1:1000; T0021, Affinity), anti‐MMP9 (1:500; AF5228, Affinity), anti‐ATF3 (1:500; DF6545, Affinity), anti‐CCL4 (1:500; D220380, Sangon Biotech), anti‐RELA (1:500; D221030, Sangon Biotech), or anti‐RELA (Phospho‐Ser536) (1:500; AF2006, Affinity) at 4°C. Finally, the second antibodies (MDL) incubate the membranes for 1 h at room temperature after washing by TBST three times. The blotted bands were detected and quantified using ChemiScope 6100 chemiluminescence imaging system (Clinx).

### 2.11 Construction of gene regulatory networks

miRNA (microRNA) is regarded as a type of noncoding RNA with a length of about 20 ~ 23 nucleotides encoded by the genome. By pairing with the mRNA bases of the target genes, they regulate posttranscriptional gene expression and either degrade mRNA or obstruct translation. TFs are a group of proteins that can selectively bind to sequences upstream of the 5′ end region of genes, increasing or inhibiting the expression of target genes with a specified intensity. Therefore, we explored whether some TFs or miRNAs in COVID‐19 and OA share a similar regulatory mechanism and developmental process targeting the hub genes. We then perform the miRNA‐gene interaction analysis based on miRTarBase (https://awi.cuhk.edu.cn/~miRTarBase/miRTarBase_2022) and TF‐gene interaction analysis based on the JASPR database (https://jaspar.genereg.net/). Gene regulatory networks, including miRNA‐ and TF‐Target gene regulatory networks were conducted and visualized using Cytoscape (Version 3.9.1).

### 2.12 Identification of candidate drugs

To identify potentially effective drugs for COVID‐19 and OA, we performed hub gene–drug interactions analysis through Drug Signatures Database (DSigDB, http://dsigdb.tanlab.org) which contains drug/gene associations based on quantitative inhibition and drug‐induced gene expression change. The drugs with adjusted *p* < .5 were identified as the candidate drugs for treatment.

### 2.13 Immune infiltration analysis

CIBERSORT is an analytical tool using linear support vector regression to deconvolute the expression matrix of different subtypes of human immune cells.^20^ It is possible to determine the presence of different immune cell subtypes in tissues by comparing the gene expression feature set of 22 immune cell subtypes and microarray expression matrix of both COVID‐19 and OA. Based on this tool, we performed the immune infiltration analysis with the R package “CIBERSORT.R.” Bar plots and heatmaps were used to display the percentage of each type of immune cell in various samples, and differences in immune cell composition between patients and controls were examined. To further identify the correlation between the content of different immune cells in GSE164805 and GSE55235, we use R package “corrplot” to perform the correlation analysis and visualize the result by heatmaps. Finally, we also used “corrplot” to identify if the hub genes were significantly associated with the content of 22 infiltrating immune cells.

### 2.14 Single‐cell analysis

The data of single‐cell RNA sequencing (scRNA‐seq) on PBMCs from COVID‐19 patients was collected from GSE171555. Meanwhile, the data of scRNA‐seq on osteoarthritic synovial membranes from OA patients were collected from GSE152805. We use R package “Seurat” to conduct quality control, expression data normalization, dimension reduction, and clustering. Cell type annotation was automatically completed by “SingleR” package. R packages “ggplot2” and “cowplot” visualized the analysis result.

## 3 RESULTS

### 3.1 Identification of the common DEGs in COVID‐19 and OA

**Figure 2:**
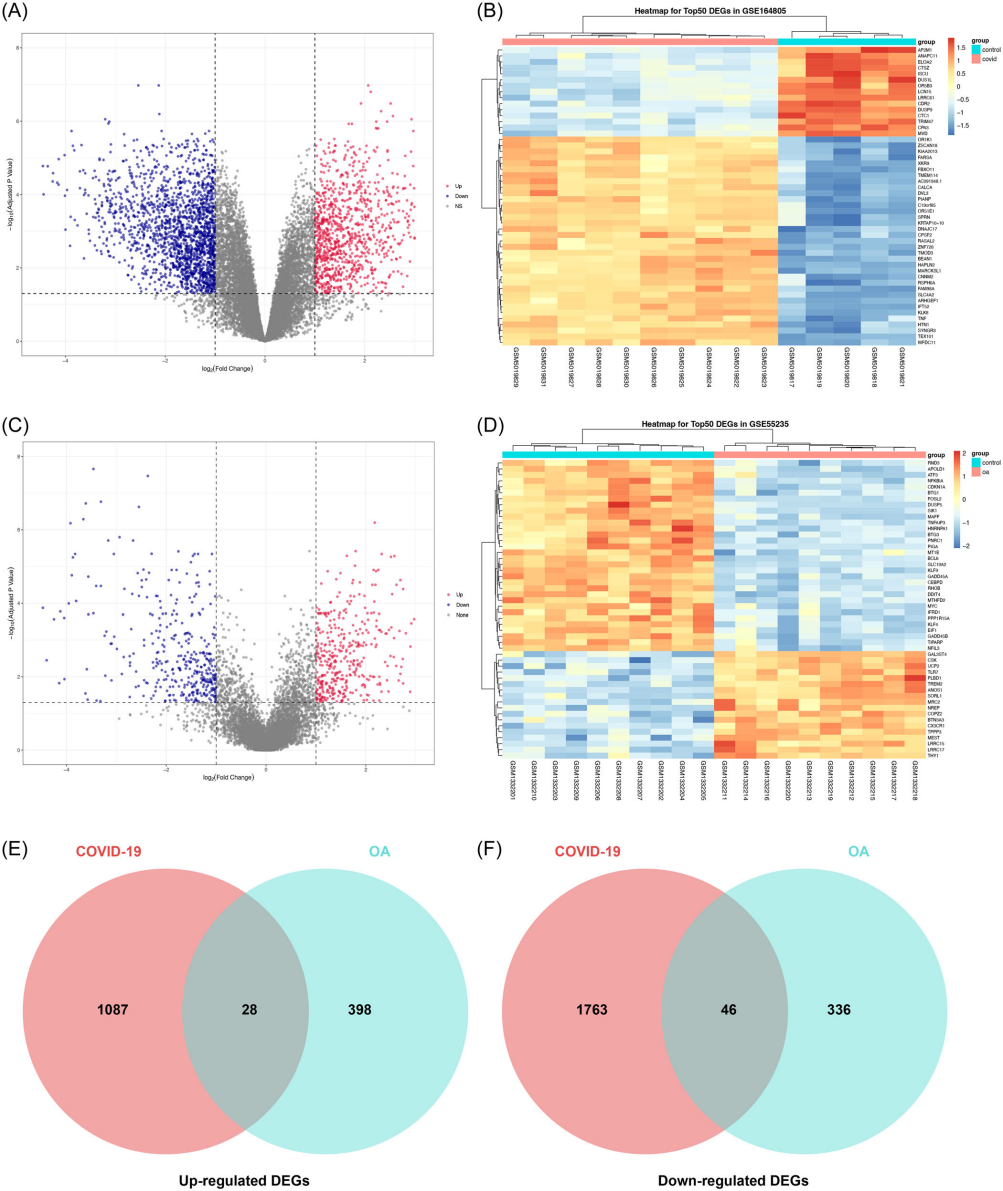
(A) Volcano plots of coronavirus disease 2019 (COVID‐19) data sets GSE164805. (B) Heatmap of the top 50 differentially expressed genes (DEGs) and 15 samples from COVID‐19 data sets GSE164805. (C) Volcano plots of osteoarthritis data sets GSE55235. (D) Heatmap of the top 50 DEGs and 20 samples from osteoarthritis data sets GSE55235. (E and F) The Venn diagram showed the common DEGs between COVID‐19 and osteoarthritis. A total of 28 genes were found common from the 1087 upregulated genes of COVID‐19 and 398 upregulated genes of osteoarthritis. A total of 46 genes were found common from the 1763 upregulated genes of COVID‐19 and 336 upregulated genes of osteoarthritis.

To identify the DEGs in COVID‐19, we first compared the mRNA expression level between the control group and the COVID‐19 patient group from GEO164805 data sets. The criteria for screening DEGs were |log2(fold change) |≥ 1 and adjusted *p* < .05. A total of 1115 upregulated and 1809 downregulated DEGs were identified and visualized by volcano plot (Figure 2A). The heatmap showed the top 50 DEGs in GEO164805 (Figure 2B). Then, we turned to find the DEGs between OA patients and healthy controls from GEO55235 data sets, while the screening criteria were the same as above. Totally 426 DEGs were significantly highly expressed while 382 DEGs showed a lower expression. The volcano plot (Figure 2C) and the heatmap (Figure 2D) presented the results. Finally, to further explore if there exists a genetic interaction between COVID‐19 and OA, we use the Venn plot to identify common DEGs between the two diseases. As is shown, 74 common genes were screened including 28 upregulated (Figure 2E) and 46 downregulated (Figure 2F) compared with the healthy people.

### 3.2 Functional enrichment analysis of the common DEGs

**Figure 3:**
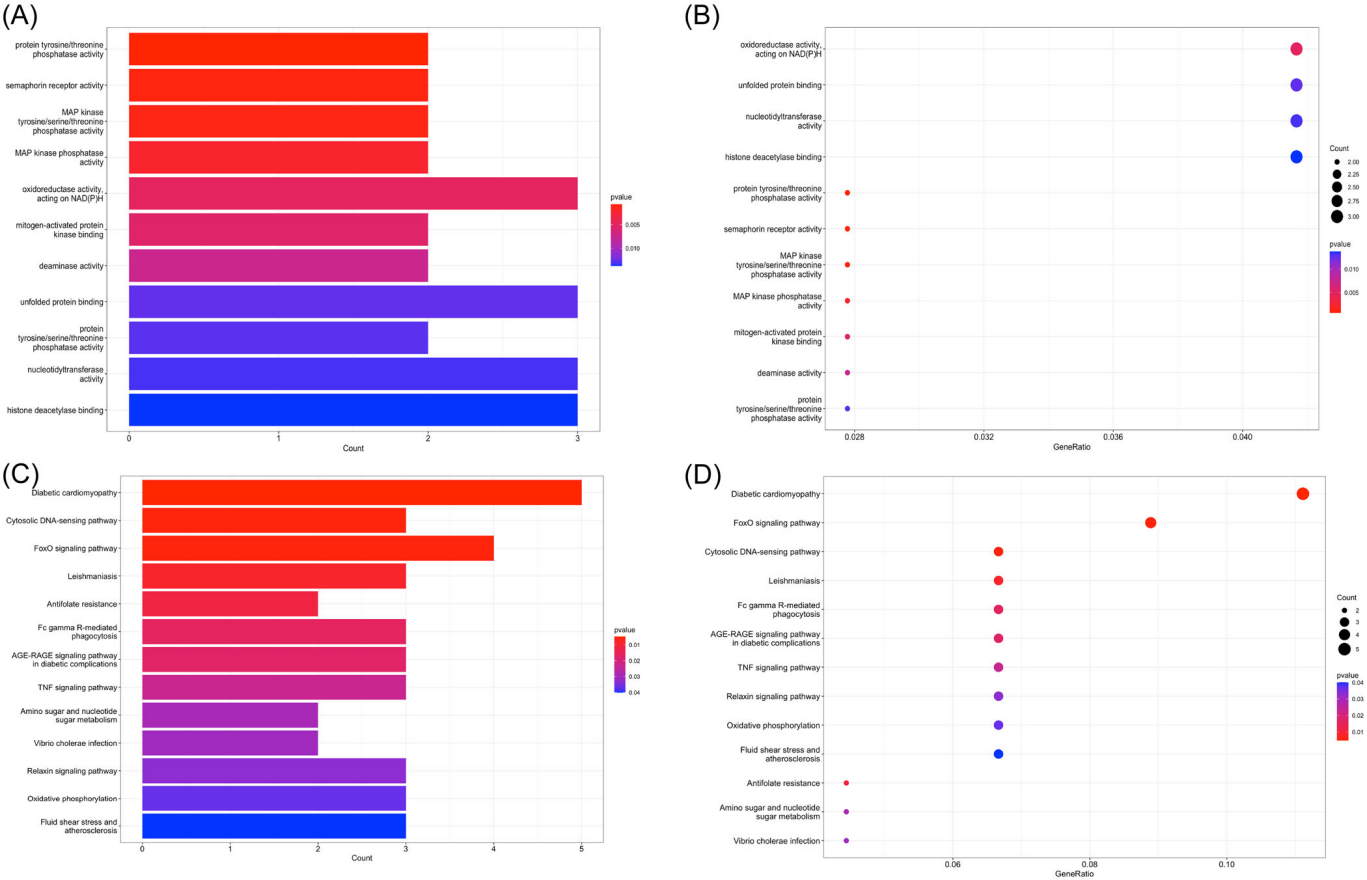
(A) Bar plot showed the results of Gene Ontology (GO) enrichment analysis of differentially expressed genes (DEGs). (B) Dot plot showed the results of GO enrichment analysis of DEGs. (C) Bar plot showed the results of Kyoto Encyclopedia of Genes and Genomes (KEGG) enrichment analysis of DEGs. (D) Dot plot showed the results of KEGG enrichment analysis of DEGs. The order of terms was arranged by *p* value in bar plots and by gene ratio in dot plots.

We next performed the functional enrichment analysis of the identified common DEGs to evaluate their potential biological effects. The results were visualized by bar plots sorted by p‐value and dot plots sorted by gene counts. As shown in Figure 3A,B, the GO enrichment analysis showed top enrich biological functions, including “protein tyrosine/threonine phosphatase activity,” “semaphoring receptor activity,” “MAP kinase tyrosine/serine/threonine phosphatase activity,” “MAP kinase phosphatase activity,” “oxidoreductase activity, acting on NAD(P)H,” “mitogen‐activated protein kinase binding,” “deaminase activity,” “unfolded protein binding,” “protein tyrosine/serine/threonine phosphatase activity,” “nucleotidyl transferase activity,” and “histone deacetylase binding.” The key pathways involving the DEGs were indicated by KEGG enrichment analysis. The results were “Diabetic cardiomyopathy,” “Cytosolic DNA‐sensing pathway,” “FoxO signaling pathway,” “Leishmaniasis,” “Antifolate resistance,” “Fc gamma R‐mediated phagocytosis,” “AGE‐RAGE signaling pathway in diabetic complications,” “TNF signaling pathway,” “Amino sugar and nucleotide sugar metabolism,” “Vibrio cholerae infection,” “Relaxin signaling pathway,” “Oxidative phosphorylation,” “Fluid shear stress and atherosclerosis” (Figure 3C,D).

### 3.3 PPI networks construction

**Figure 4:**
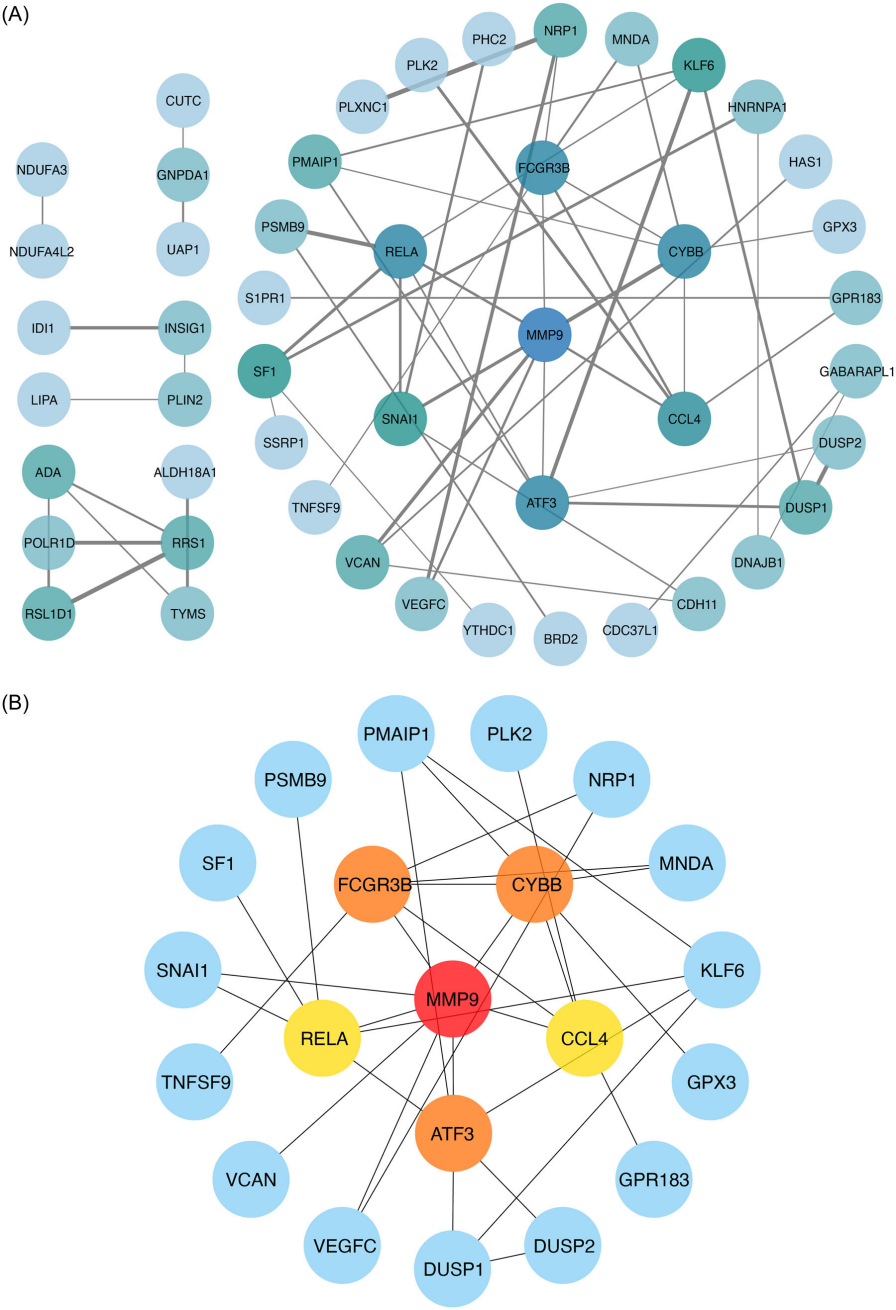
Construction of protein–protein interaction network (A) and identification of the top six hub genes (B) of differentially expressed genes common to coronavirus disease 2019 and osteoarthritis. Nodes represent proteins and the edges show the relationships between proteins. Nodes with darker colors have more important roles.

We use the STRING database to find the PPI between the common DEGs. As mentioned above, 74 common genes were identified and 48 of them were predicted to have interaction and were visualized with the PPI networks (Figure 4A). Genes with a darker color were more dominant in the PPI networks, which may indicate that they play a more significant role in COVID‐19 and OA.

### 3.4 Identification and functional enrichment analysis of the hub genes

**Figure 5:**
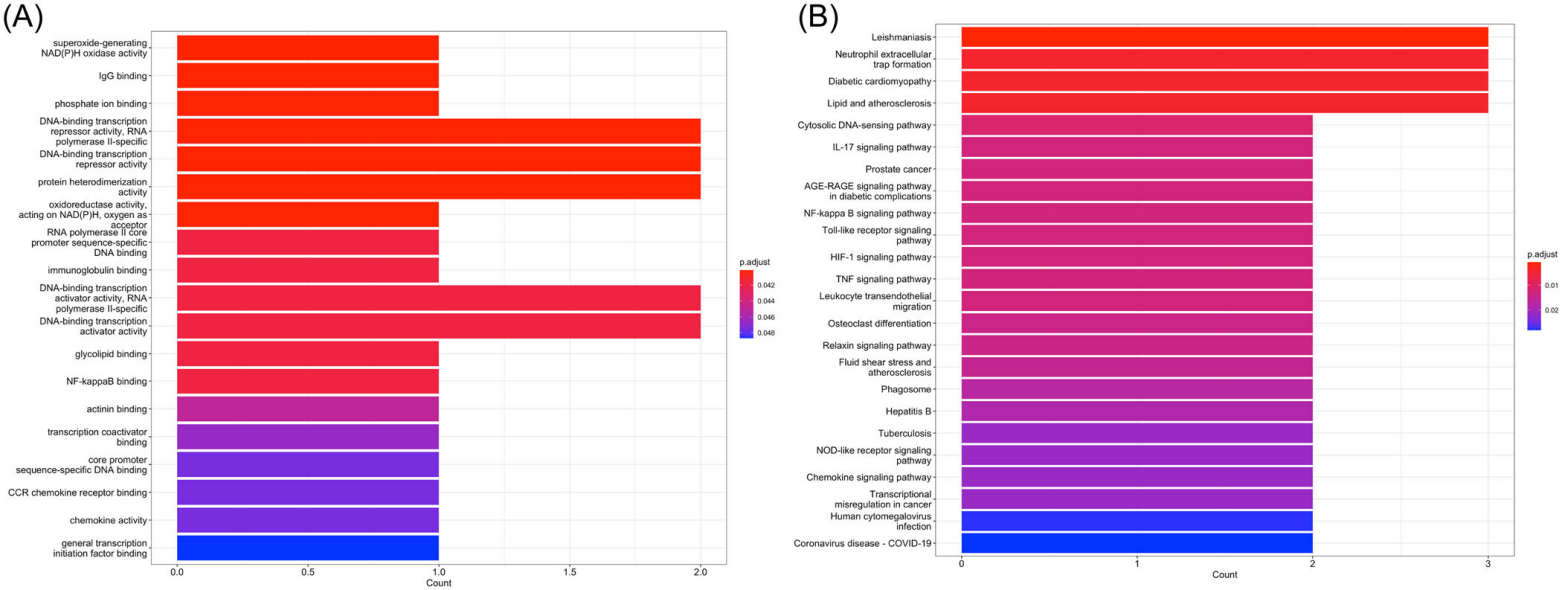
(A) Bar plot showed the results of Gene Ontology enrichment analysis of six hub genes. (B) Bar plot showed the results of Kyoto Encyclopedia of Genes and Genomes enrichment analysis of six hub genes. The order of terms was arranged by *p* value.

The MCC method of CytoHubba was widely used for hub gene identification. According to the scores given, we choose the top six genes with scores >8 as the hub genes, including MMP9, ATF3, FCGR3B, CYBB, CCL4, and RELA. Then based on the six hub genes, we constructed the PPI networks with 15 genes which were all highly associated with the hub genes (Figure 4B). GO enrichment analysis of the six hub genes resulted in 19 biological effects with *p* < .05, including “superoxide‐generating NAD(P)H oxidase activity,” “IgG binding,” “phosphate ion binding,” “DNA‐binding transcription repressor activity, RNA polymerase II‐specific,” “DNA‐binding transcription repressor activity,” “oxidoreductase activity acting on NAD(P)H, oxygen as acceptor,” “RNA polymerase II core promoter sequence‐specific DNA binding,” “immunoglobulin binding,” “DNA‐binding transcription activator activity, RNA polymerase II‐specific,” “DNA‐binding transcription activator activity,” “glycolipid binding,” “NF‐kappa B binding,” “actinin binding,” “transcription coactivator binding,” “core promoter sequence‐specific DNA binding,” “CCR chemokine receptor binding,” “chemokine activity,” and “general transcription initiation factor binding” (Figure 5A). We also performed KEGG enrichment analysis and the top pathways related to six hub genes were “Leishmaniasis,” “Neutrophil extracellular trap formation,” “Diabetic cardiomyopathy,” “Lipid and atherosclerosis,” “Cytosolic DNA‐sensing pathway,” “interleukin (IL)‐17 signaling pathway,” “Prostate cancer,” “AGE‐RAGE signaling pathway in diabetic complications,” “NF‐kappa B signaling pathway,” “Toll‐like receptor signaling pathway,” “HIF‐1 signaling pathway,” “TNF signaling pathway,” “Leukocyte transendothelial migration,” “Osteoclast differentiation,” “Relaxin signaling pathway,” “Fluid shear stress and atherosclerosis,” “Phagosome,” “Hepatitis B,” “Tuberculosis,” “NOD‐like receptor signaling pathway,” “Chemokine signaling pathway,” “Transcriptional misregulation in cancer,” “Human cytomegalovirus infection,” and “Coronavirus disease—COVID‐19” (Figure 5B).

### 3.5 The diagnostic value and external validation of the hub genes

**Figure 6:**
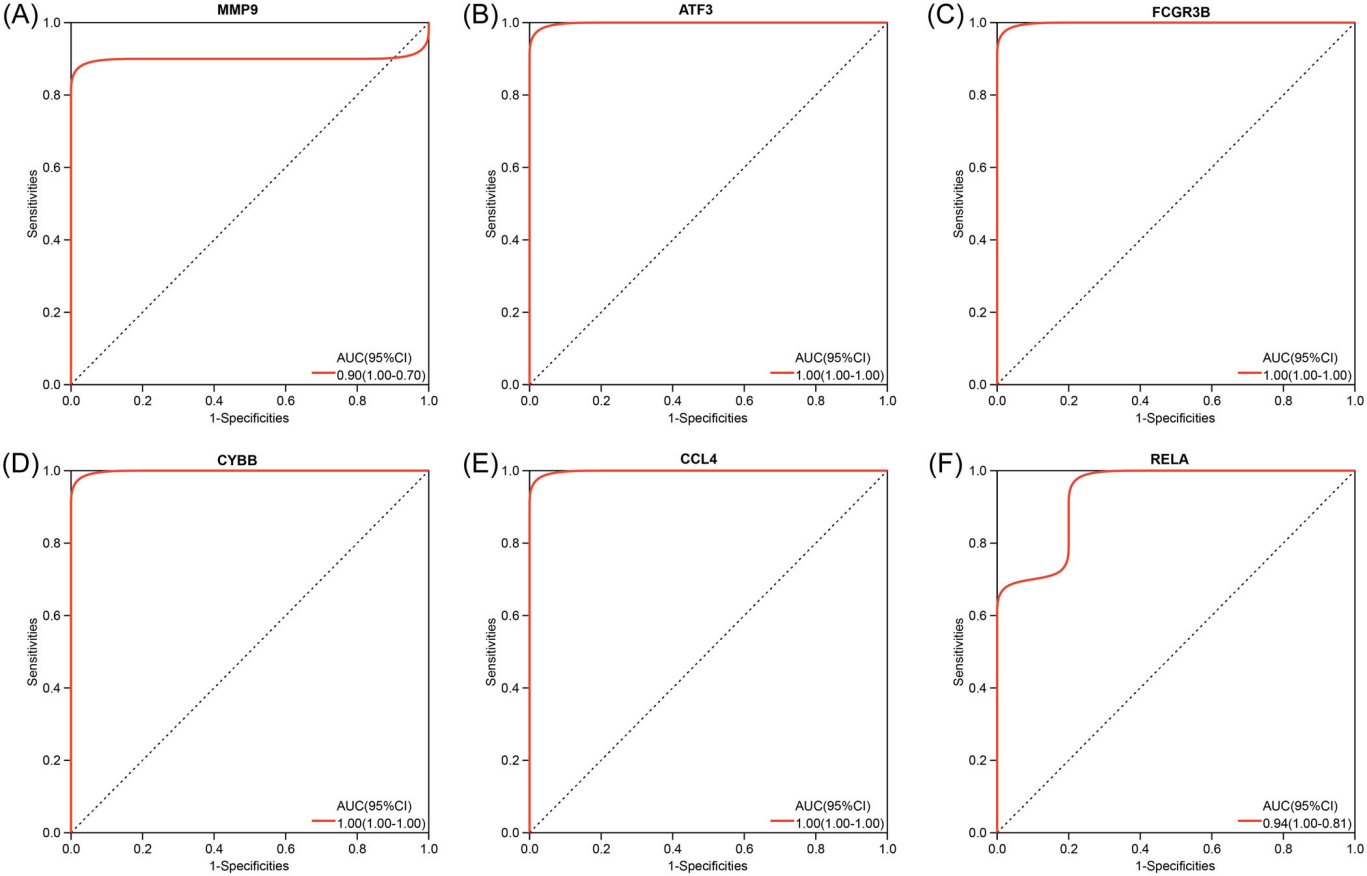
The hub genes' diagnostic value in coronavirus disease 2019 (COVID‐19) data sets GSE164805. Receiver operating characteristic curves and area under curve statistics are used to evaluate the capacity of MMP9 (A), ATF3 (B), FCGR3B (C), CYBB (D), CCL4 (E), and RELA (F) to discriminate COVID‐19 from healthy controls with excellent sensitivity and specificity.

**Figure 7:**
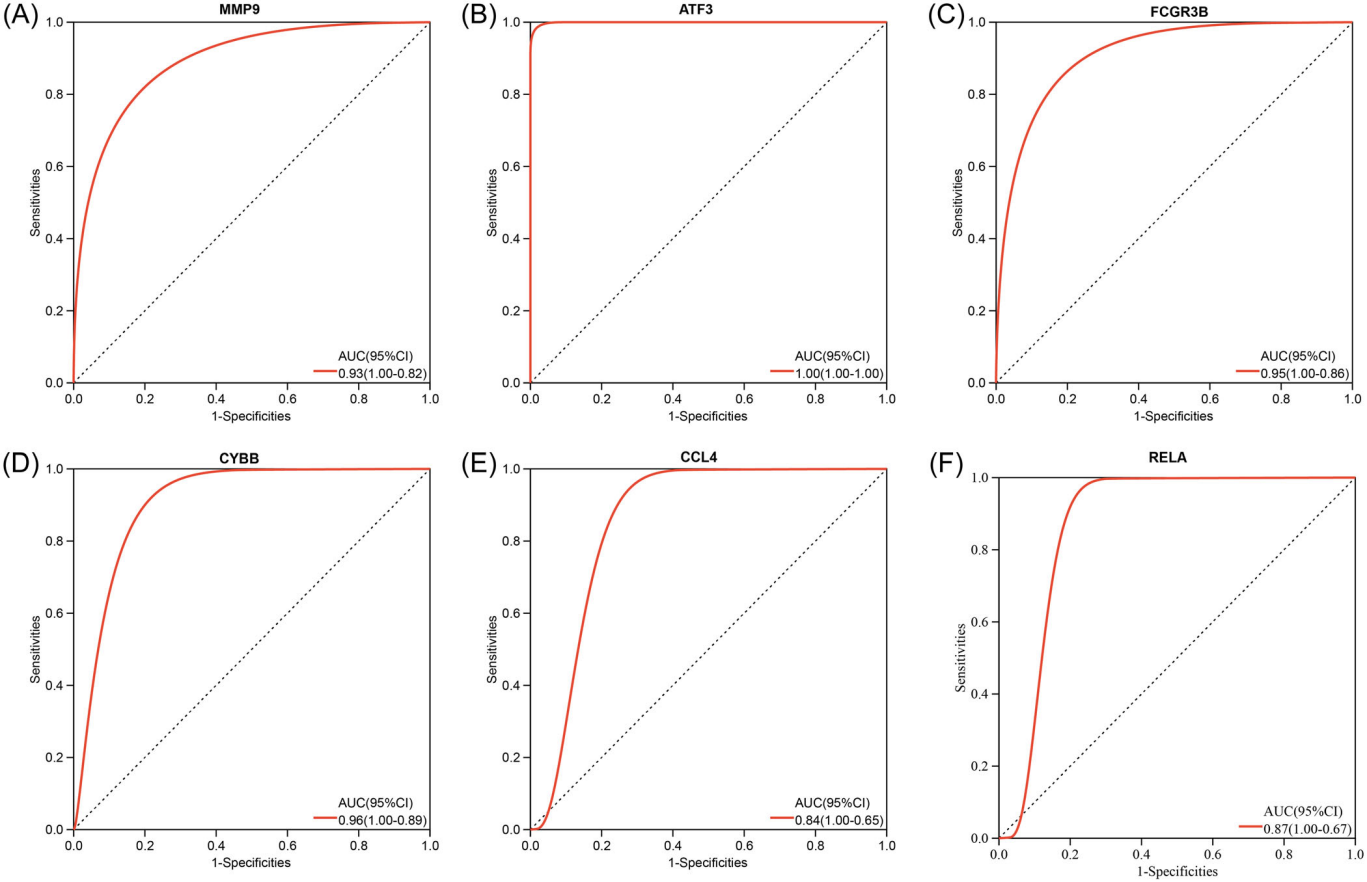
The hub genes' diagnostic value in osteoarthritis data sets GSE55235. Receiver operating characteristic curves and area under curve statistics are used to evaluate the capacity of MMP9 (A), ATF3 (B), FCGR3B (C), CYBB (D), CCL4 (E), and RELA (F) to discriminate osteoarthritis from healthy controls with excellent sensitivity and specificity.

**Figure 8:**
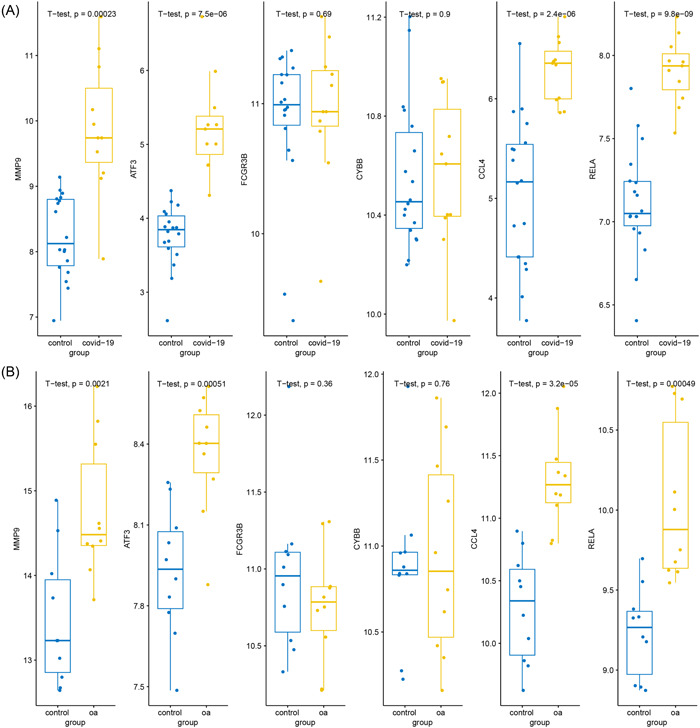
(A) Box plots showed the expression of six hub genes in GSE177477 (validation data sets of coronavirus disease 2019). (B) Box plots showed the expression of six hub genes in GSE117999 (validation data sets of osteoarthritis). *p < .05, **p < .01, ***p < .001.

**Figure 9:**
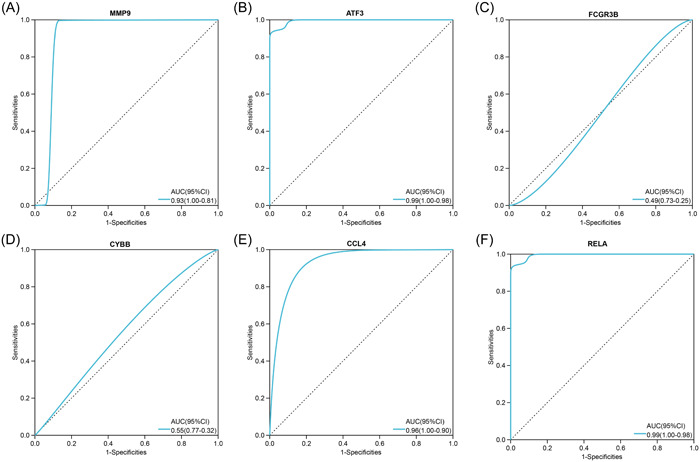
Validation of the hub genes' diagnostic value in coronavirus disease 2019 (COVID‐19) data sets GSE177477. Receiver operating characteristic curves and area under curve (AUC) statistics are used to evaluate the capacity of MMP9 (A), ATF3 (B), FCGR3B (C), CYBB (D), CCL4 (E), and RELA (F) to discriminate COVID‐19 from healthy controls with excellent sensitivity and specificity. Four (matrix metalloproteinase 9, ATF3, CCL4, RELA) of the six hub genes have good diagnostic capacity with AUC > 0.7.

**Figure 10:**
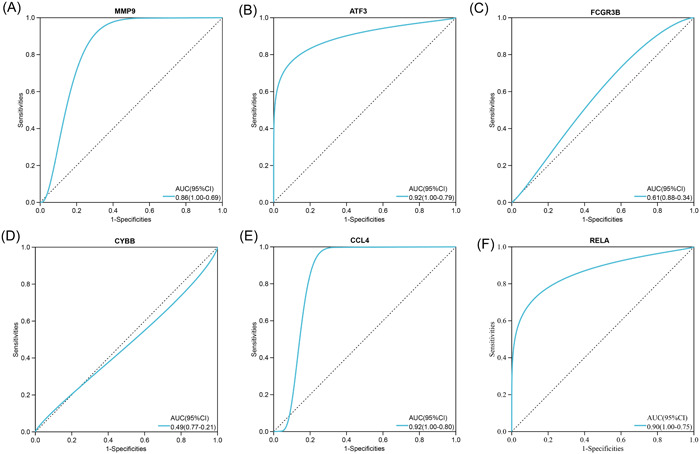
Validation of the hub genes' diagnostic value in osteoarthritis data sets GSE117999. Receiver operating characteristic curves and area under curve (AUC) statistics are used to evaluate the capacity of MMP9 (A), ATF3 (B), FCGR3B (C), CYBB (D), CCL4 (E), and RELA (F) to discriminate osteoarthritis from healthy controls with excellent sensitivity and specificity. Four (matrix metalloproteinase 9, ATF3, CCL4, RELA) of the six hub genes have good diagnostic capacity with AUC > 0.7.

We conducted ROC analyses and calculated the AUC values which reveal the diagnostic value of the hub genes. As shown in Figure 6, in the training data set GSE164805 of COVID‐19, all six hub genes (MMP9, ATF3, FCGR3B, CYBB, CCL4, and RELA) showed a significant diagnostic value with high AUC values (0.90, 1.00, 1.00, 1.00, 1.00 and 0.94, respectively). For the OA data set GSE55235, six hub genes also have good diagnostic effects with the AUC value = 0.93, 1.00, 0.95, 0.96, 0.84, and 0.87 respectively (Figure 7). To further validate the hub genes' role in COVID‐19 and OA, we downloaded GSE17747 as the external validation data set of COVID‐19 and GSE117999 as the external validation data set of OA. In both data sets, MMP9, ATF3, CCL4, and RELA showed significantly higher expression in patients than in controls (*p* < .05) while FCGR3B and CYBB had no difference between the two populations (Figure 8). ROC analyses further confirmed the results that MMP9, ATF3, CCL4, and RELA had great diagnostic abilities in COVID‐19 with AUC > 0.7 (AUC = 0.93, 0.99, 0.96, and 0.99) (Figure 9). Likewise, the AUC values of four hub genes in OA were 0.86, 0.92, 0.92, and 0.90, which indicated the great diagnostic role of MMP9, ATF3, CCL4, and RELA in OA (Figure 10).

### 3.6 Validation of the identified hub genes in the OA mouse model

**Figure 11:**
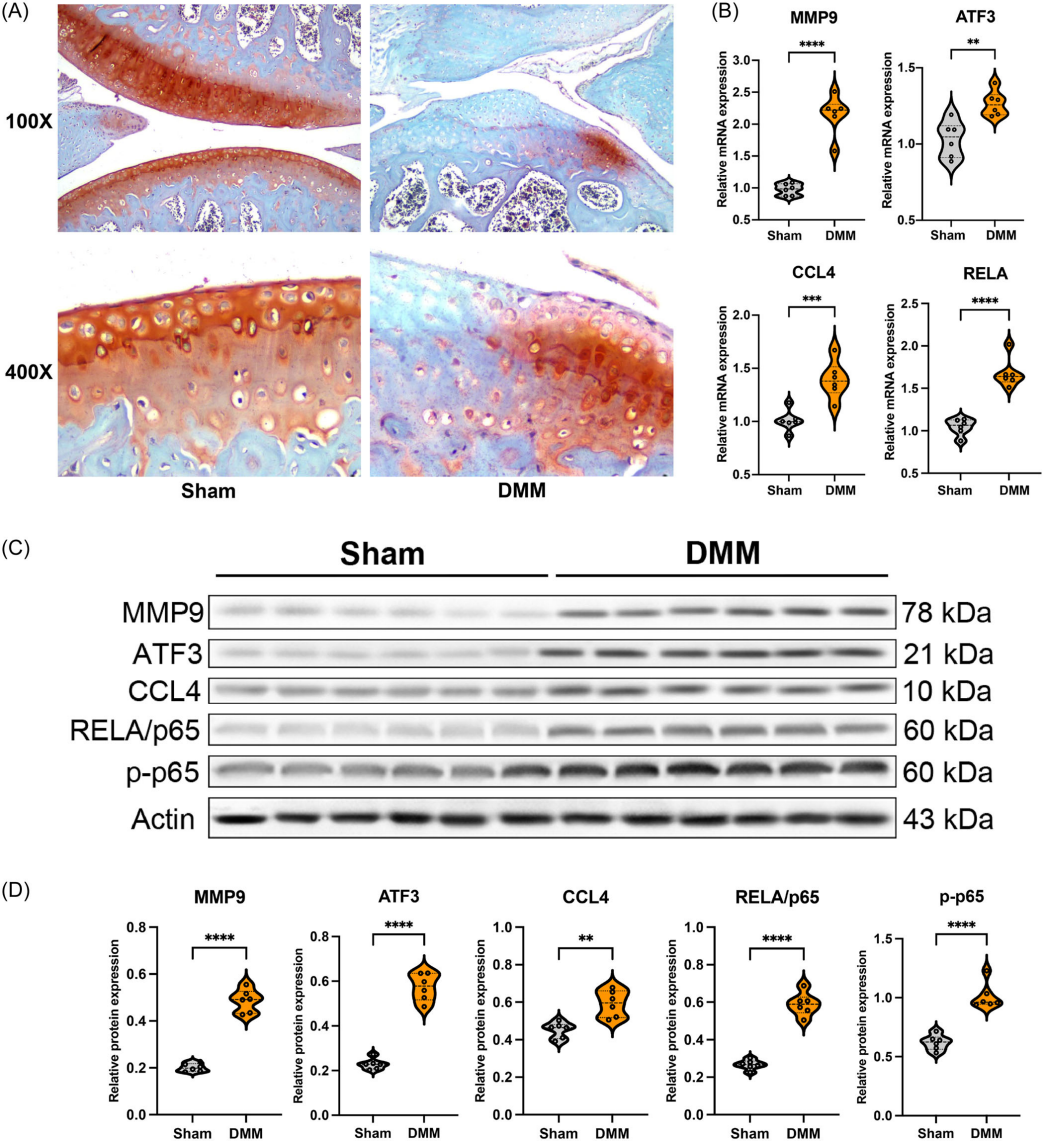
Four hub genes (matrix metalloproteinase [MMP] 9, ATF3, CCL4, RELA) were upregulated in destabilization of the medial meniscus (DMM) mouse model. (A) Safranine O‐Fast Green staining of cartilage in Sham and DMM group 4 weeks after surgery (100X and 400X). (B) The relative mRNA expression of MMP9, ATF3, CCL4, and RELA in mice cartilage tissues of each group. (C) The protein expression of MMP9, ATF3, CCL4, RELA/p65, and p‐p65 in mice cartilage tissues of each group. (D) Quantification of relative protein expression by Western Blot.

OA mouse model was induced by DMM surgery, and the control group was induced by Sham surgery. After 4 weeks, the knee joints were stained by safranin O‐fast green, which made the cartilage tissues red and bone tissues blue. As the 100X and 400X staining images shown in Figure 11A, the cartilage of DMM‐induced mice showed significant degeneration compared with the Sham group, as well as the structure in the region became disordered, which met the OA phenotypes. The relative mRNA expression of the four hub genes (MMP9, ATF3, CCL4, and RELA) in cartilage tissues was measured by qPCR. All the hub genes in the DMM groups were upregulated in mRNA levels (Figure 11B). Moreover, Western Blot further confirmed the higher protein expression of MMP9, ATF3, CCL4, RELA/p65, and p‐p65 in the DMM group than in the Sham group (Figure 11C,D).

### 3.7 Interaction analysis of DEGs‐miRNAs and DEGs‐TFs

**Figure 12:**
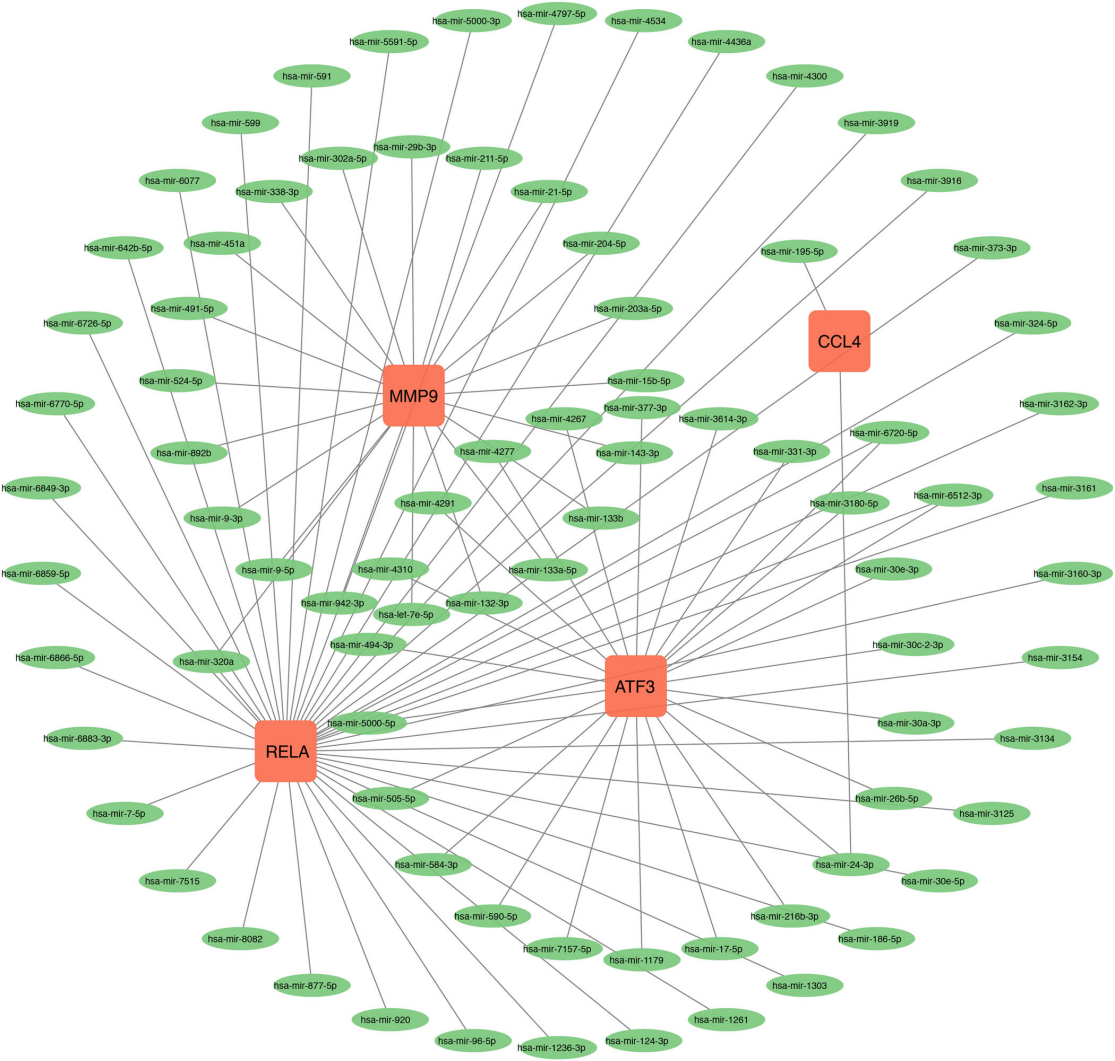
Interconnected regulatory interaction network of differentially expressed genes–microRNAs. There are 88 nodes and 90 edges in the network.

**Figure 13:**
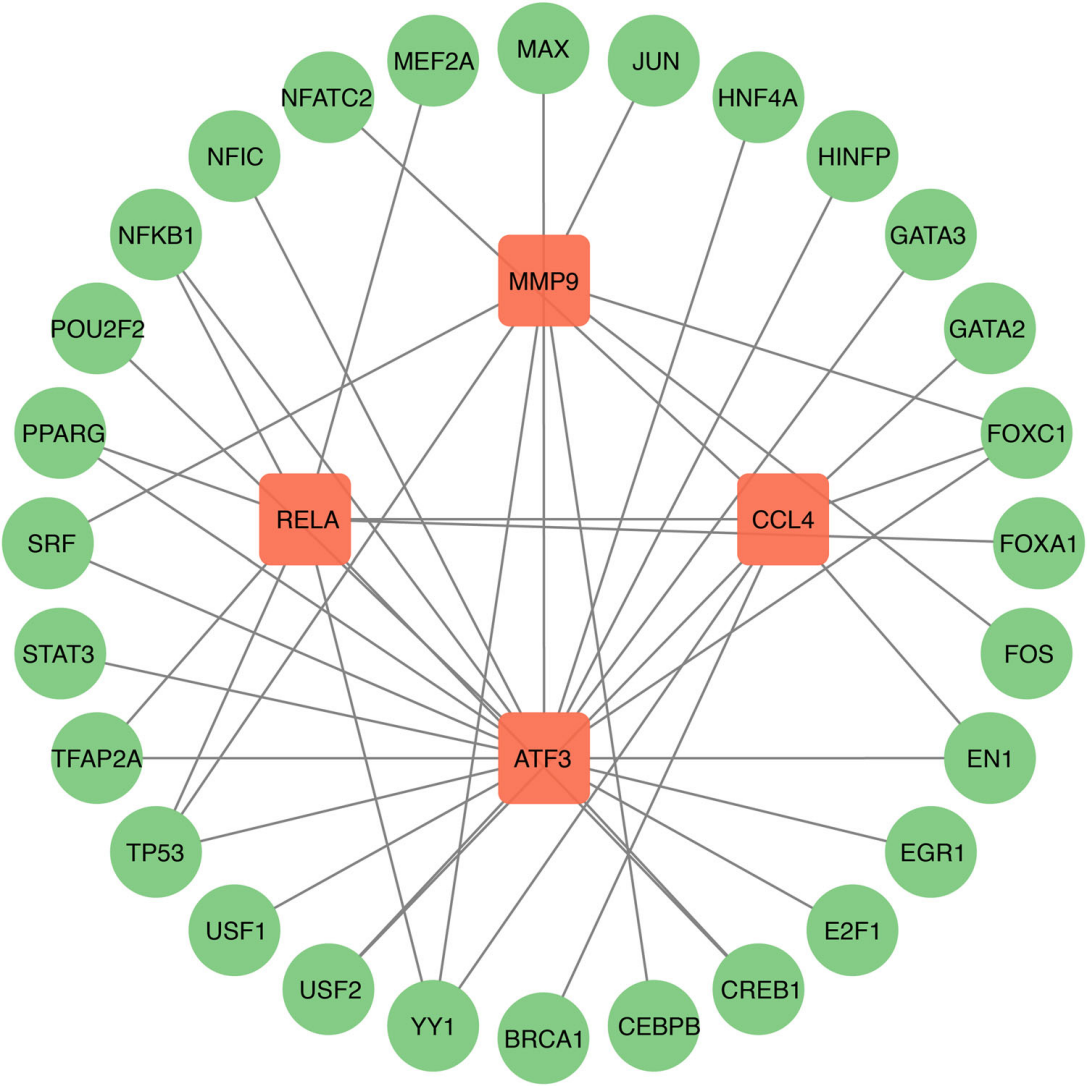
Interconnected regulatory interaction network of differentially expressed genes–transcriptional factors. There are 32 nodes and 41 edges in the network.

As miRNAs and TFs have been proven, an important role in regulating the transcription process and protein expression, which may positively or negatively influence the genes' function in diseases, we performed the interaction analysis between the identified four hub genes and miRNAs or TFs. Finally, 84 miRNAs and 28 TFs were identified to be associated with four hub genes, and the DEGs‐miRNA (Figure 12) and DEGs–TFs (Figure 13) interaction networks were constructed.

### 3.8 Prediction of the candidate drugs

Gene–drug interaction analysis can predict the potential small molecule drugs that target gene‐related diseases. Here, we searched the DSigDB database based on the four identified hub genes and found drugs that may have great effects on both COVID‐19 and OA. Drugs were sorted by adjusted *p* < .05 and the top 10 candidate drugs were “2‐Mercaptobenzothiazole,” “Simvastatin,” “DL‐Mevalonic acid,” “Hydrocortisone,” “Nickel sulfate,” “Dinoprostone,” “Deoxynivalenol,” “Troglitazone,” “Acrolein,” and “Thalidomide” (Table 1). The chemical formula and structure of candidate drugs are also shown in Table 1.

**Table 1:** The candidate drugs for COVID‐19 and OA.

Name	Chemical formula	*p* Value	Adjusted *p* value	Structure
2‐Mercaptobenzothiazole	C_7_H_5_NS_2_	4.86E‐08	2.73E‐05	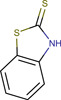
Simvastatin	C_25_H_38_O_5_	5.23E‐08	2.73E‐05	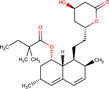
DL‐Mevalonic acid	C_6_H_12_O_4_	1.31E‐07	4.54E‐05	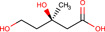
Hydrocortisone	C_2_H_30_O_5_	4.41E‐07	1.15E‐04	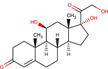
Nickel sulfate	NiO_4_S	6.81E‐07	1.41E‐04	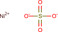
Dinoprostone	C_20_H_32_O_5_	9.96E‐07	1.41E‐04	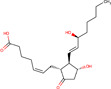
Deoxynivalenol	C_15_H_20_O_6_	1.07E‐06	1.41E‐04	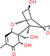
Troglitazone	C_24_H_27_NO_5_S	1.11E‐06	1.41E‐04	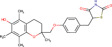
Acrolein	C_3_H_4_O	1.31E‐06	1.41E‐04	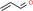
Thalidomide	C_13_H_10_N_2_O_4_	1.37E‐06	1.41E‐04	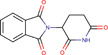

Abbreviations: COVID‐19, coronavirus disease 2019; OA, osteoarthritis.

### 3.9 Immune infiltration analysis and the correlation between genes and immune cells

**Figure 14:**
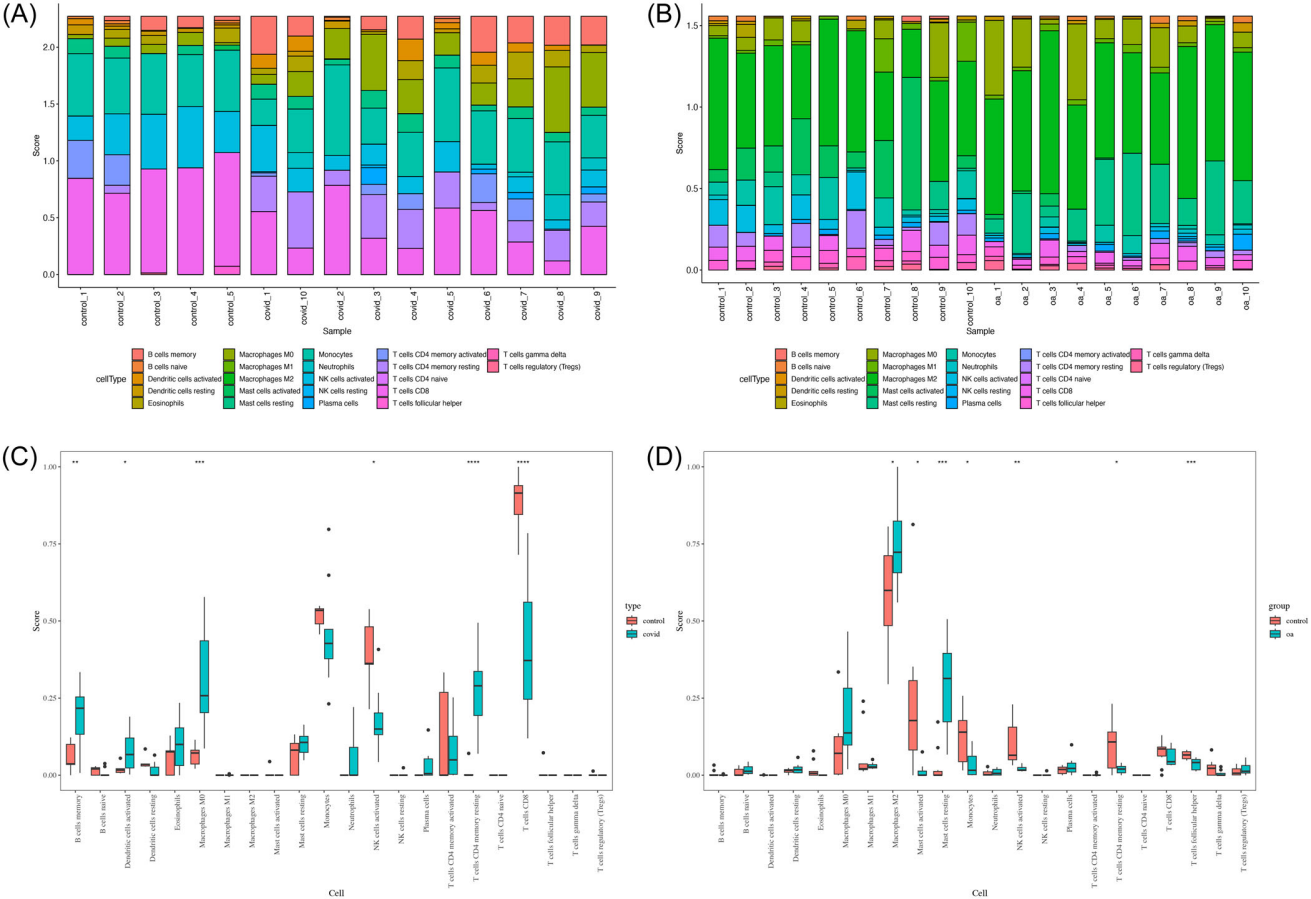
Immune cell infiltration analysis on coronavirus disease 2019 (COVID‐19) and osteoarthritis. The proportion of 22 kinds of immune cells in COVID‐19 patients and controls visualized from the box plot (A) and the bar plot (C). The proportion of 22 kinds of immune cells in osteoarthritis patients and controls was visualized from the box plot (B) and the bar plot (D). *p < .05, **p < .01, ***p < .001.

**Figure 15:**
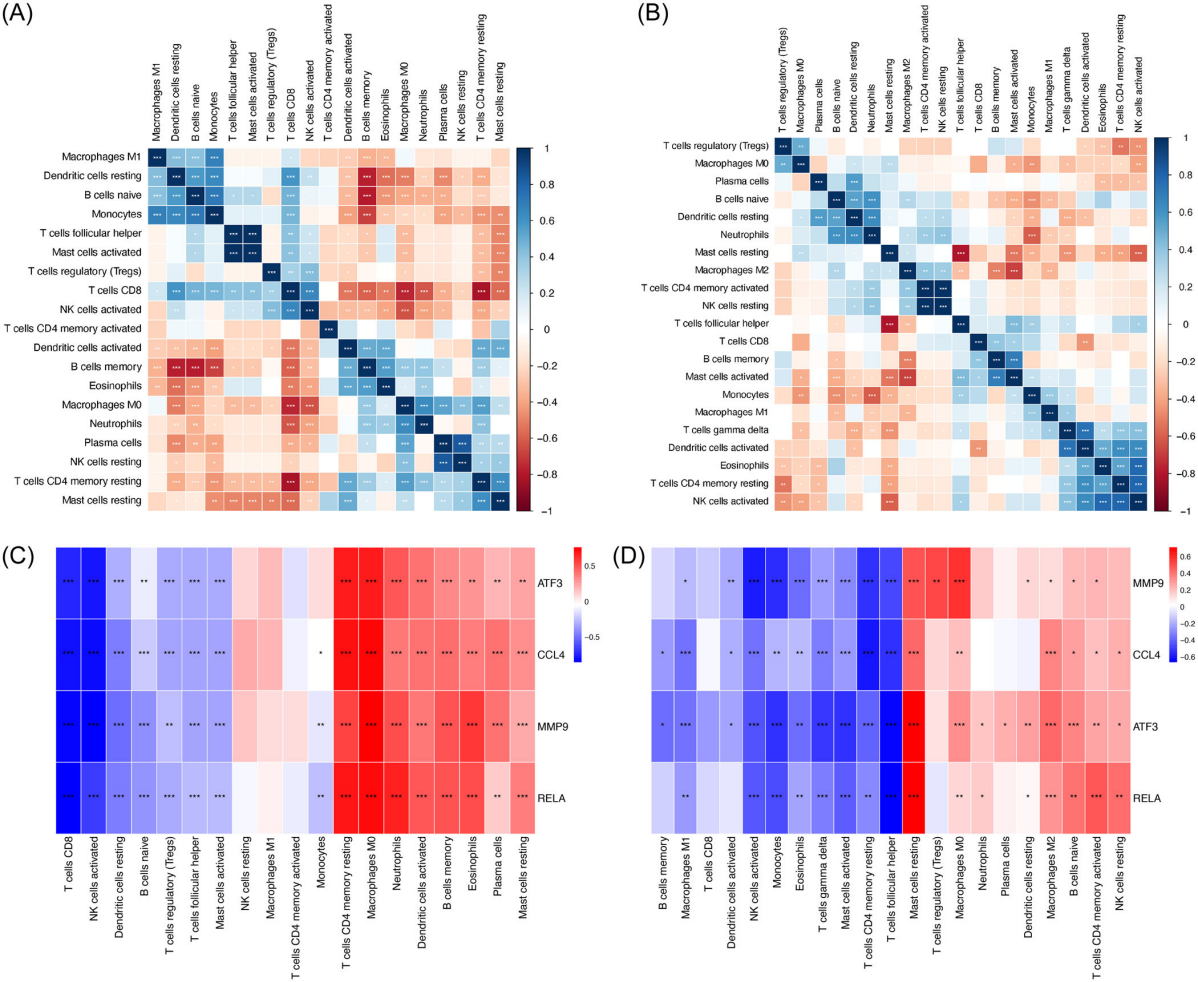
Correlation of 22 immune cell type compositions in coronavirus disease 2019 (COVID‐19) (A) and osteoarthritis (B). Correlation between the expression of 22 immune cells and four hub genes in COVID‐19 (C) and osteoarthritis (D). *p < .05, **p < .01, ***p < .001.

Since both COVID‐19 and OA were highly associated with the immune system, we were interested in the immune infiltration levels in these two diseases. The relative content of 22 types of immune cells was calculated based on the mRNA expression matrix (GSE164805 and GSE55235) and CIBERSORT tools. The bar charts showed the immune cell composition in each sample in healthy controls and COVID‐19 or OA patients (Figure 14A,B). Then, we compared the relative scores of different immune cells in COVID‐19 and OA by the linear regression algorithm of CIBERSORT. As shown in Figure 14C,B cells memory (*p* < .01), Dendritic cells activated (*p* < .05), Macrophages M0 (*p* < .001), and T cells CD4 naïve (*p* < .0001) were higher expressed in COVID‐19 patients than in healthy controls, while Natural killer (NK) cells activated (*p* < .05) and T cells CD8 (*p* < .0001) were downregulated in COVID‐19. In OA patients, Macrophage M2 (*p* < .05), Mast cells resting (*p* < .001) were more than the controls, and Mast cells activated (*p* < .05), Monocytes (*p* < .05), NK cells activated (*p* < .01), T cells CD4 memory resting (*p* < .05), and T cells follicular helper (*p* < .001) had higher expression in the OA group compared with the control group (Figure 14D). We then evaluated the correlation between 22 different immune cell subtypes in COVID‐19 patients. B cells memory was also positively correlated with Neutrophils, Eosinophils, and Plasma cells while it had a negative correlation with Monocytes and T cells CD8 (Figure 15A). Dendritic cells activated and Macrophages M0 showed similar results with B cells memory (Figure 15A). Both NK cells activated and T cells CD8 were positively correlated with Dendritic cells resting and Mast cells activated and negatively correlated with Dendritic cells activated, B cells memory, Eosinophils, Macrophages M0, Neutrophils, and Plasma cells (Figure 15A). As for OA patients, Macrophages M2 had a positive correlation with B cells naïve, Neutrophils, Mast cells resting, T cells CD4 memory activated, and NK cells resting and was negatively related with Mast cells activated and Macrophages M1 (Figure 15B). Monocytes in OA were correlated with Mast cells activated and Macrophages M1 positively while with Macrophages M0, B cells naïve, Neutrophils, and Macrophages M2 negatively (Figure 15B). Finally, the correlation between four hub genes and immune cell expression was shown in Figure 15C of COVID‐19 and in Figure 15D of OA. Interestingly, all four genes were positively correlated with Macrophage M0 and negatively correlated with NK cells activated and Mast cells activated in COVID‐19 and OA (Figure 15C,D).

### 3.10 Single‐cell analysis

**Figure 16:**
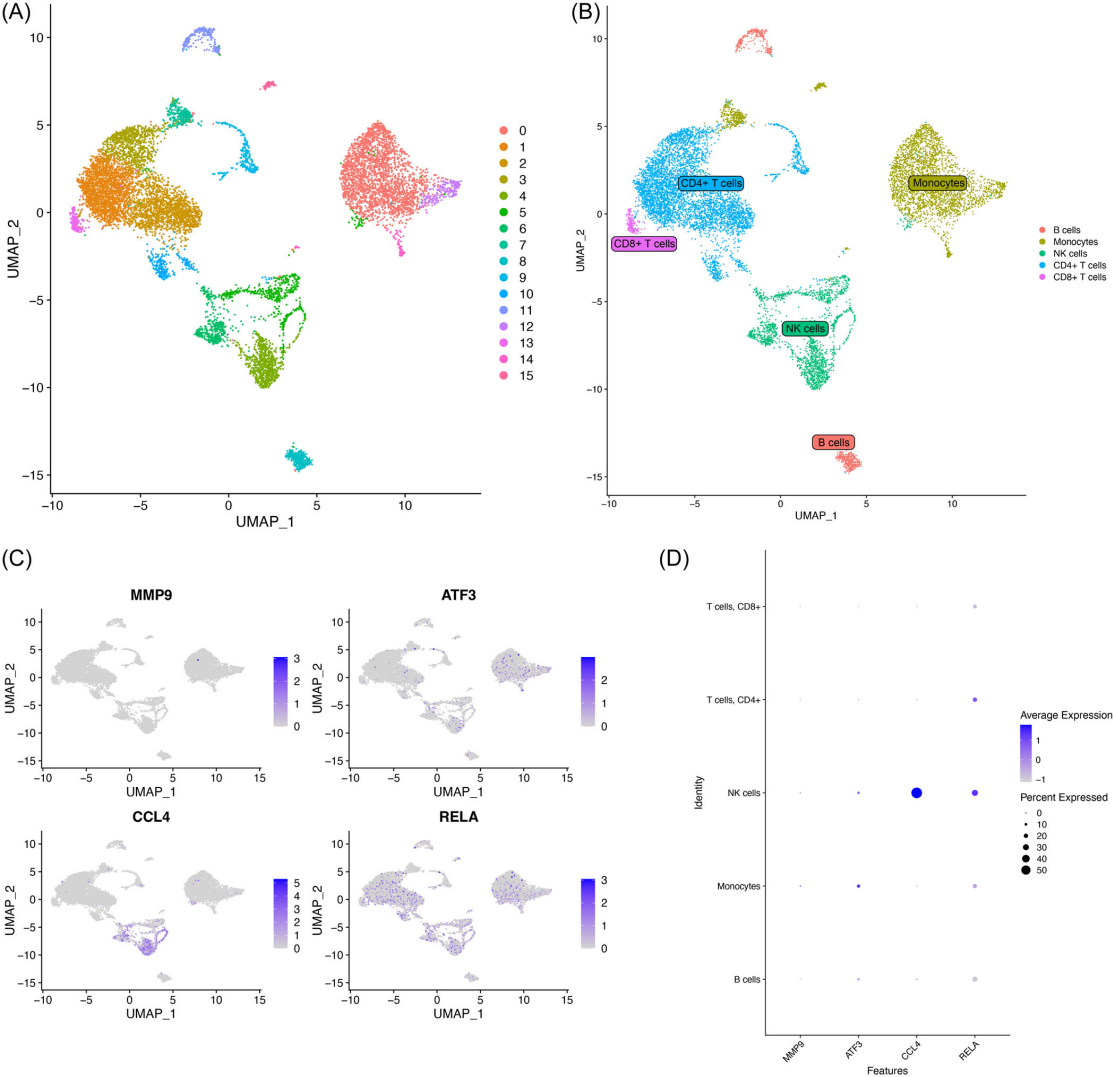
Single‐cell RNA analysis on peripheral blood mononuclear cells (PBMCs) from coronavirus disease 2019 (COVID‐19) patients. (A) UMAP plot showed 15 clusters of PBMCs from COVID‐19 patients. (B) UMAP distributions of single cells from the five defined cell types annotated by SingleR. Feature plot (C) and dot plot (D) showed the expression of four hub genes in identified clusters and cell types. UMAP, uniform manifold approximation and projection.

**Figure 17:**
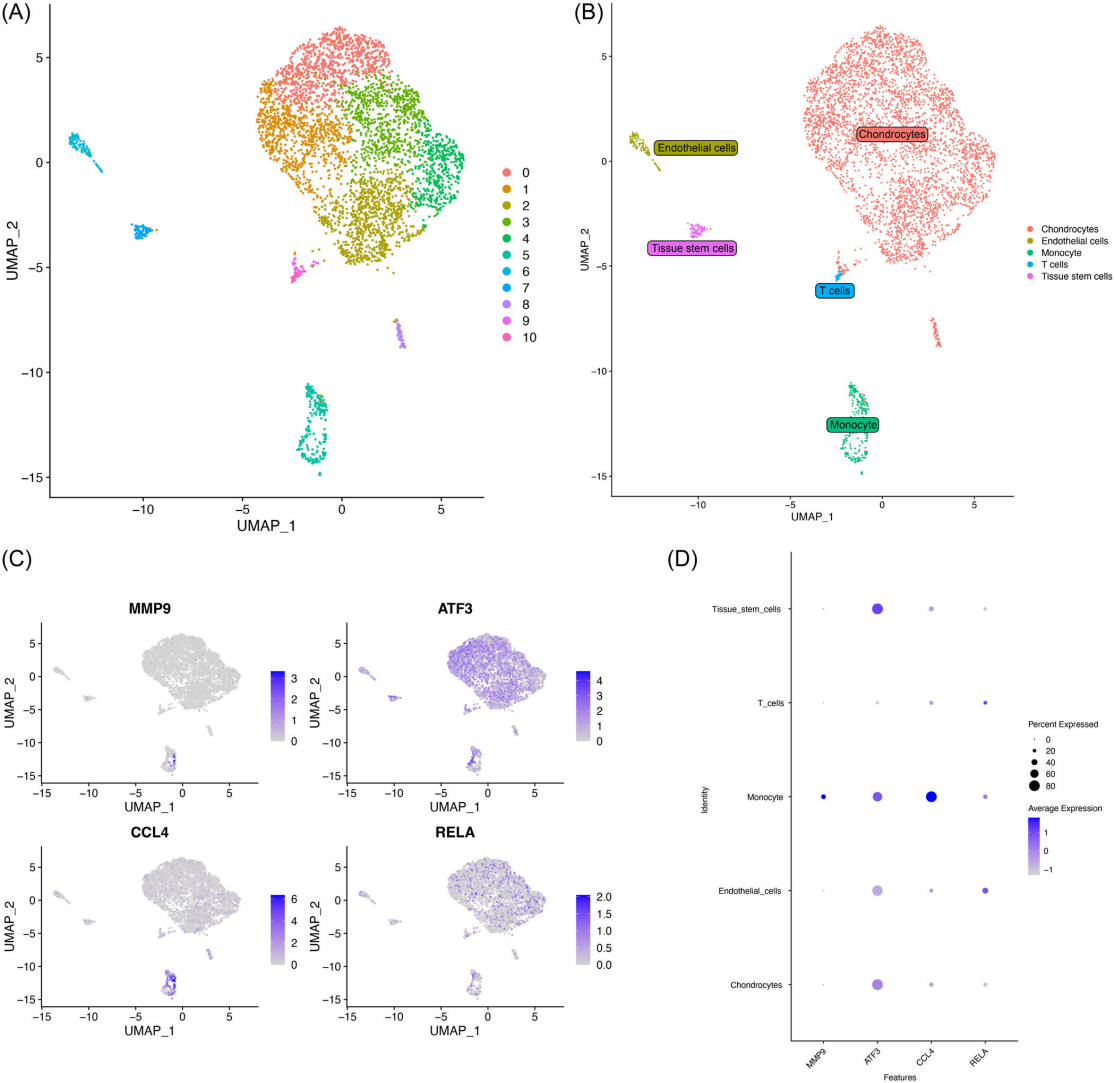
Single‐cell RNA analysis on osteoarthritic synovial membrane from osteoarthritis patients. (A) UMAP plot showed 10 clusters of osteoarthritic synovial membrane from coronavirus disease 2019 patients. (B) UMAP distributions of single cells from the five defined cell types annotated by SingleR. Feature plot (C) and dot plot (D) showed the expression of four hub genes in identified clusters and cell types. UMAP, uniform manifold approximation and projection.

The scRNA‐seq data of COVID‐19 was from the PBMCs of COVID‐19 patients. The uniform manifold approximation and projection (Figure 16A) showed the 15 clusters of whole cells after dimension and clustering. We then used the “SingleR” package to annotate the 15 clusters into biological cell types, and the five clusters were B cells, Monocytes, NK cells, CD4 + T cells, and CD8 + T cells (Figure 16B). The distribution of four hub genes (MMP9, ATF3, CCL4, RELA) in each cluster was shown in Figure 16C by feature plot. As shown in Figure 16D, ATF3, CCL4, and RELA were highly expressed in NK cells while ATF3 had higher expression in Monocytes and RELA had more expression in CD4 + T cells. sc‐RNA sequencing data of OA was obtained from the synovial tissue in OA patients and was analyzed by the same process as COVID‐19 data. A total of 10 clusters were shown (Figure 17A) and then five types of cells were identified including Chondrocytes, Endothelial cells, Monocyte, T cells, and Tissue stem cells (Figure 17B). The feature plot showed the distribution of the four hub genes (Figure 17C). MMP9 and CCL4 were mainly expressed in Monocytes while RELA showed higher expression in Endothelial cells and T cells (Figure 17D).

## 4 DISCUSSION

The COVID‐19 pandemic has affected more than a billion people worldwide and is still evolving, and the powerful variability of the virus has led researchers to become increasingly interested in specific treatments for COVID‐19. It is worth noting that COVID‐19 affects older people more than other populations, with severe and fatal COVID‐19 often occurring in older people with or without underlying medical conditions. OA used to be thought of as a degenerative joint disease common in the elderly that worsened with age. However, several studies have shown that the occurrence and development of OA may be related to systemic inflammation and immune system dysfunction, which are the key pathogenesis of COVID‐19. Meanwhile, epidemiological studies have shown an increased prevalence and severity of musculoskeletal disorders such as OA in people with COVID‐19, especially in the elderly. The co‐occurrence of COVID‐19 and OA may bring society a huge burden on economic and medical resources. In this study, we used bioinformatics and animal experimental methods to identify four common genes of COVID‐19 and OA, and then conducted a series of bioinformatics analyses to bring new perspectives on potential diagnostic and therapeutic strategies for comorbidity of COVID‐19 and OA.

We identified 74 common DEGs of COVID‐19 and OA. GO enrichment analysis was performed on the DEGs to reveal the possible pathophysiology of these two diseases. Protein phosphatase activity and MAP kinase phosphatase activity were among the top GO terms. The enzyme activity of the PTP SH2 domain‐containing phosphatase 2 (SHP‐2) was significantly increased in human OA samples with serious articular cartilage injury and in IL‐1β‐stimulated mouse chondrocytes.^21^ A recent study further found that SHP099, an allosteric inhibitor of SHP‐2, attenuated OA progression by inhibiting M1 macrophage polarization.^22^ As for COVID‐19, Genome‐wide association studies found that alkaline phosphatase was associated with severe COVID‐19^23^ while proteomics analysis in plasma further showed a higher expression of protein phosphatase in recovered COVID‐19 patients than that of healthy subjects.^24^ Virus infection‐related diseases, including COVID‐19, would activate the p38 mitogen‐activated protein kinase (MAPK) signaling pathway, and using SB203580, an inhibitor of p38, can attenuate MAPK activity and resist SARS‐CoV‐2.^25^ Moreover, MAPK was also related to inflammation, which was the key pathogenesis of OA.^26^ A study has shown that Synovial fluid from end‐stage OAs could induce the proliferation and fibrosis of articular chondrocytes via MAPK signaling.

Diabetic cardiomyopathy and FoxO signaling pathway were the top enriched terms by KEGG analysis. Several studies have reported that cardiomyopathy could be seen in acute COVID‐19 patients and was considered an important complication in hospitals.^27^, ^28^, ^29^ Meanwhile, it was noticed that mice with type 2 diabetes had more severe symptoms of COVID‐19^30^ and evidence suggested a higher risk of diabetes after SARS‐CoV‐2 infection.^31^, ^32^ However, there is no evidence that diabetic cardiomyopathy is associated with OA to date, so further research is warranted. FoxO TFs also played an important role in both COVID‐19^33^ and OA.^34^, ^35^ Therefore, targeting FoxO signaling may help treat the co‐occurrence of these two diseases.

PPI network based on DEGs was conducted to identify key features of common proteins and then help explore potential therapeutic strategies. We screened six top hub genes (MMP9, ATF3, FCGR3B, CYBB, CCL4, and RELA) that were highly associated with both COVID‐19 and OA by the MCC method. Expression data and ROC analysis further validated the hub genes in both training data sets and external validation data sets of COVID‐19 and OA, and four genes (MMP9, ATF3, CCL4, RELA) had higher expression in patients than healthy people with great diagnostic values. We further conducted OA mouse models by DMM surgery, and the results showed that the RNA and protein expression of MMP9, ATF3, CCL4, and RELA was significantly higher in the cartilage tissue of OA mice, which was consistent with the validation results.

MMPs were a type of enzyme that could degrade almost all protein components in the extracellular matrix (ECM) and were essential for the destruction of articular cartilage and the pathogenesis of OA.^36^ In OA patients, MMP9 could be secreted by chondrocytes and osteoclasts to destroy cartilage and inhibit the formation of blood vessels.^36^ It was also reported that MMP9‐mediated Syndecan‐4 shedding promoted the deterioration of OA.^37^ Moreover, MMP9 was identified as a biomarker of early respiratory failure in COVID‐19 patients because of its role in ECM remodeling.^38^ Also, higher MMP9 level in blood was positively correlated with mortality in COVID‐19 patients.^39^

ATF3 was a TF that was involved in ferroptosis^40^ and endoplasmic reticulum stress,^41^ influencing the pathogenesis of various diseases. It has been found that nasopharyngeal samples and nasal epithelial cells of COVID‐19 patients had higher ATF3 expression.^42^, ^43^ Moreover, ATF3 was upregulated by inflammatory cytokines in the OA cartilage of both mice and humans through the nuclear factor‐kB pathway.^44^ ATF3 can also regulate the activation of MMPs in OA chondrocytes,^45^ indicating the potential interaction between the identified hub genes (MMP9 and ATF3) in OA development.

CCL4, also called macrophage inflammatory protein 1 beta, is a chemokine secreted by activated monocytes and involved in a variety of immune and inflammatory responses. Research showed that CCL4 was excessively released by Macrophages or T cells in bronchoalveolar lavage fluid and PBMCs of COVID‐19 patients.^46^, ^47^ Resistin binds CAP1 and upregulates the expression of proinflammatory cytokines (including CCL4) and MMPs via p38‐MAPK and NF‐κB signaling in OA chondrocytes.^48^ By contrast, in OA mice, miR‐495 promoted chondrocyte apoptosis and inhibited its proliferation by reducing the expression of CCL4 and NF‐κB signaling.^49^ Further studies should be conducted to reveal the exact role of CCL4 in OA.

RELA, or p65, was the key component of the NF‐κB signaling pathway and was involved in various important physiological and pathological processes such as inflammation, stress, and apoptosis. Targeting the p65‐NF‐κB signaling pathway has been proven greatly essential for antivirus and anti‐inflammation in COVID‐19.^50^, ^51^, ^52^ Moreover, the activity of NF‐κB signaling in OA chondrocytes promoted proteasome degradation and secretion of MMPs, resulting in the development and deterioration of OA phenotypes.^53^ In the pathogenesis of patients with comorbidity of COVID‐19 and OA, RELA and NF‐κB signaling may play a pivotal role, which may be of great help in diagnosis and treatment.

miRNAs were highly conserved and involved in posttranscriptional gene regulation. Binding with mRNA via RNA‐silencing, miRNAs could inhibit the expression of target genes and further influence the development of diseases. We conducted the miRNA–gene interaction network and four miRNAs (hsa‐mir‐320a, hsa‐mir‐6512‐3p, hsa‐mir‐6720‐5p, and hsa‐mir‐24‐3p) could bind with more than two hub genes as the prediction. Next‐generation sequencing analysis demonstrated that miR‐320a deeply decreased in COVID‐19 patients with severe respiratory failure.^54^ Moreover, miR‐320a had higher expression in OA chondrocytes and promote matrix degeneration^55^ while a study conversely found that miR‐320a upregulated the expression of RUNX2 and inhibited OA cartilage degeneration.^56^ Machine Learning predicted that miR‐24‐3p, targeting NRP‐1, significantly decreased in COVID‐19 patients^57^ and could also inhibit S protein expression and SARS‐CoV‐2 replication.^58^ As for OA, the chondrocyte destruction induced by IL‐1β was attenuated by miR‐24‐3p,^59^ further indicating that miR‐24‐3p may alleviate the severity of both COVID‐19 and OA.

TFs are essential for various cell functions and disease development. They can bind to promoter regions located upstream of gene coding regions, activating or inhibiting gene transcription, which determines whether the gene functions. Here we predicted 28 TFs that may bind with identified hub genes. Among them, FOXC1, TP53, and YY1 show the highest degree. The overexpression of FOXC1 in synovial fibroblasts promoted the pathology of OA^60^ while both miR‐138‐5p and miR‐204‐5p could attenuate OA inflammation and cartilage degeneration by targeting FOXC1.^61^, ^62^ SGT‐53, a novel drug carrying a plasmid vector driving expression of the human TP53 gene (encoding p53), activated the antivirus immune reaction and was the potential therapy for COVID‐19.^63^ Meanwhile, lncRNA H19 reduces inflammation and ameliorates OA‐related cartilage damage and chondrocyte apoptosis by activating TP53.^64^ It has been proved that YY1 plays an important role in cancers and rheumatoid arthritis (RA). Through the activation of Th17 differentiation, YY1 is essential for IL‐6 transcription in RA, which contributes to RA inflammation.^65^ However, no abundant evidence showed the role of YY1 in COVID‐19 or OA and further studies should be conducted to find the potential therapy for these two diseases targeting YY1.

We then predicted the candidate drugs of COVID‐19 and OA based on the four hub genes and selected the top 10 significant drugs by adjusted *p* value. Simvastatin can reduce cholesterol levels in the blood has been approved by the Food and Drug Administration and is widely used for lipid‐lowering and various cardiovascular diseases. By lipid raft disruption, Simvastatin had the functions of antivirus and anti‐inflammation and showed early protective effects on COVID‐19.^66^ Also, Simvastatin inhibited the endothelial activation induced by the SARS‐CoV‐2 nucleocapsid protein.^67^ Another common drug was Hydrocortisone, which had a long history of treating immune disorders and inflammation. Clinical studies demonstrated some positive effects of Hydrocortisone on COVID‐19^68^ and OA,^69^ but the great adverse effects avoided its wide use on nonsevere patients. Troglitazone was a peroxisome proliferator‐activated receptor γ (PPARγ) agonist and insulin sensitizer, mainly used in the treatment of type 2 diabetes mellitus and insulin resistance. By inhibiting the nonstructural proteins 9 of SARS‐Cov‐2, troglitazone could prevent virus replication and become the treatment option for COVID‐19.^70^ PPARγ deficiency led to severe OA phenotypes in mice,^71^, ^72^ which indicates that troglitazone may help attenuate OA severity by activating PPARγ.

SARS‐Cov‐2 infection activates the body's immune response and causes disorders of the immune system like cytokine storm which induces whole‐body manifestations and makes it difficult to treat severe cases of COVID‐19.^73^ Although OA was not defined as an immune‐related disease and was different from RA traditionally, recent studies demonstrated that the immune reaction was involved in OA pathogenesis, and this was also confirmed by elevated levels of immunoglobulin and complement in the synovial fluid of the joints in patients with OA.^74^, ^75^ In this study, immune infiltration levels of COVID‐19 and OA were evaluated by CIBERSORT, and the results showed that Macrophages were increased while NK cells and Mast cells were decreased in both these two diseases. Four hub genes (MMP9, ATF3, CCL4, RELA) were all significantly correlated with the immune cell expression and single‐cell analysis confirmed the results. It was widely reported that macrophage infiltration occurred in patients' airways and would lead to lung injury.^76^, ^77^ NLRP3 inflammasome, which was the key regulator to activate body macrophage reaction, played an important role in COVID‐19 and the potential clinical treatment targeting it is being studied.^78^ Moreover, OA development was associated with macrophage polarization and macrophage was transformed from resting to activating. In this process, macrophage M1 that promotes inflammation would increase while the number of anti‐inflammation macrophage M2 would reduce.^79^ Many biomaterials have been produced and applied based on the mechanism of macrophage in OA.^80^, ^81^ Mast cells degranulation could be induced by SARS‐CoV‐2 in airway inflammation, leading to lung injury in COVID‐19.^82^ Moreover, the activation of mast cells existed in postacute COVID‐19 syndrome^83^ and Long‐COVID.^84^ Some studies indicated that patients with OA were found to have a higher number of mast cells and more degranulation.^85^, ^86^ However, mast cells showed anti‐inflammation effects during OA development by secreting mediators that inhibit the function of IL.^87^, ^88^

There are also some limitations in our study. First, the sample size was not large enough although we used the mRNA microarray and single‐cell sequencing data sets and validated it in the external data set and mouse model. Second, the model of COVID‐19 was hard to construct because the SARS‐CoV‐2 must be obtained and studied in a biological safety third‐level laboratory. Therefore, a large‐scale clinical trial should be needed to further validate the role of hub genes in COVID‐19 and OA, and ensure the safety and efficacy of candidate drugs.

## 5 CONCLUSIONS

In this study, four key hub genes (MMP9, ATF3, CCL4, RELA) showed higher expression and effective diagnostic ability in the co‐occurrence of COVID‐19 and OA. Disease‐related miRNAs, TFs, drugs, and immune infiltration help to understand the mechanisms of pathogenesis and offer a strong theoretical foundation for the future development of diagnostic methods and molecular therapies for the co‐occurrence of COVID‐19 and OA.

## AUTHOR CONTRIBUTIONS

Xuhui Zhou designed the process of the study. Bowen Lai and Heng Jiang analyzed and interpreted the sequencing data and were major contributors in writing the manuscript. Bowen Lai and Taotao Liao performed animal experiments. Yuan Gao and Xuhui Zhou improved the writing of the manuscript. All authors read and approved the final manuscript.

## CONFLICT OF INTEREST STATEMENT

The authors declare no conflict of interest.

## ETHICS STATEMENT

All animals were kept in an SPF environment. All animal experiments were approved and conducted with the guidelines of the Ethics Committee on Animal Experiments of the Second Military Medical University.

## Data Availability

All data generated or analyzed during this study are included in this published article and its supplementary information files. Other necessary data are available from the corresponding author on reasonable request.
